# Morphological and Phylogenetic Evidences Reveal Four New Species of *Cantharellus* Subgenus *Cantharellus* (Hydnaceae, Cantharellales) From China

**DOI:** 10.3389/fmicb.2022.900329

**Published:** 2022-06-27

**Authors:** Yu-Zhuo Zhang, Wen-Fei Lin, Bart Buyck, Zhi-Qun Liang, Ming-Sheng Su, Zuo-Hong Chen, Ping Zhang, Shuai Jiang, Dong-Yu An, Nian-Kai Zeng

**Affiliations:** ^1^Key Laboratory of Tropical Translational Medicine of Ministry of Education, School of Pharmacy, Hainan Medical University, Haikou, China; ^2^College of Science, Hainan University, Haikou, China; ^3^Institute of Edible and Medicinal Fungi, College of Life Sciences, Zhejiang University, Hangzhou, China; ^4^UMR 7205, Institut Systématique, Evolution, Biodiversité, Muséum National d’Histoire Naturelle, Sorbonne Université, CNRS, Paris, France; ^5^Key Laboratory of Prevention and Treatment of Cardiovascular and Cerebrovascular Diseases of Ministry of Education, Gannan Medical University, Ganzhou, China; ^6^College of Life Science, Hunan Normal University, Changsha, China; ^7^School of Pharmaceutical Sciences and Yunnan Key Laboratory of Pharmacology for Natural Products, Kunming Medical University, Kunming, China; ^8^Yinggeling Substation, Hainan Tropical Rainforest National Park, Baisha, China

**Keywords:** chanterelle, molecular phylogeny, morphology, new taxa, taxonomy

## Abstract

Species of *Cantharellus* subgenus *Cantharellus* are interesting and important for their mycorrhizal properties, medicinal values, and edibility. In China, there are many undescribed species of the subgenus. In this study, four new species of subg. *Cantharellus*, *viz. Cantharellus albopileatus*, *Cantharellus chuiweifanii*, *Cantharellus pinetorus*, and *Cantharellus ravus* from Hainan and Hunan Provinces, respectively, were described based on morphological and phylogenetic evidence as a contribution to the knowledge of the species diversity in China. Detailed descriptions, color photographs of fresh basidiomata, and line drawings of microstructures of these four new species are presented as well as comparisons with related species.

## Introduction

Species diversity, taxonomy, and phylogeny of macrofungi have been investigated in the recent years, and many new species have been discovered (Zeng et al., 2013; Han et al., 2016; Chai et al., 2019; Cui et al., 2019; Shen et al., 2019; Sun et al., 2020; Liu et al., 2021a,b, 2022; Wu et al., 2021; Ji et al., 2022; Xie et al., 2022). Species of *Cantharellus* Adans. ex Fr. (Hydnaceae, Cantharellales), interesting and important fungi, have also received a lot of attention by mycologists for their mycorrhizal properties, medicinal values, and edibility (Pilz et al., 2003; Yun and Hall, 2004; Shao et al., 2012). Molecular phylogeny has delimited abundant species within the genus and revealed unexpected species diversity (Buyck et al., 2011, 2013, 2014, 2016a,b; Foltz et al., 2013; Leacock et al., 2016). Until now, a large number of *Cantharellus* taxa have been described in Europe, Africa, and North America (Corner, 1966; Buyck et al., 2011, 2013, 2014, 2016a,b, 2018; Kumari et al., 2011, 2013; Suhara and Kurogi, 2015; De Kesel et al., 2016; Leacock et al., 2016).

**FIGURE 1:**
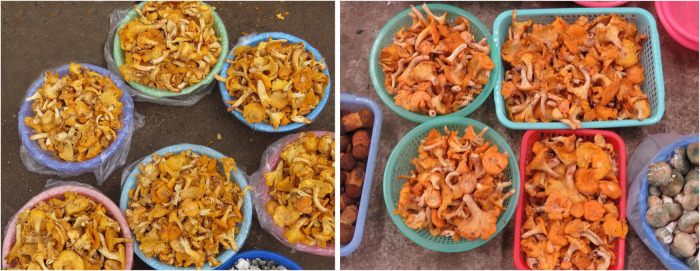
Collections of chanterelle sold as edibles in the market of Yunnan Province, southwestern China. Photographs were taken by N. K. Zeng.

In China, many species of *Cantharellus* have been uncovered (Chiu, 1973; Zang, 1980; Wei et al., 2008; Tian et al., 2009, 2012; Shao et al., 2011, 2012, 2014, 2016a,^2021^; Buyck et al., 2018; An et al., 2017; Jian et al., 2020; Cao et al., 2021; Zhang M. et al., 2021). They are well known to the public in the country because most of them have activities of anticancer, antimicrobial, immune regulation, and antioxidant (Dulger et al., 2004; Daniewskia et al., 2012; Nowacka-Jechalkea et al., 2018; Zhao et al., 2018). Interestingly, basidiocarps of *Cantharellus* such as *Cantharellus yunnanensis* W. F. Chiu are sold as edibles in markets in Yunnan Province, southwestern China (Figure 1), which generate good economic value (Watling, 1997; Wu and Lu, 2006).

Recently, the genus *Cantharellus* has been divided into six subgenera including subg. *Afrocantharellus* Eyssart. and Buyck, subg. *Cantharellus* Adans. ex Fr., subg. *Cinnabarinus* Buyck and V. Hofst., subg. *Parvocantharellus* Eyssart. and Buyck, subg. *Pseudocantharellus* Eyssart. and Buyck, and subg. *Rubrinus* Eyssart. and Buyck (Buyck et al., 2014). Among them, subg. *Cantharellus* has received considerable attention, which is characterized by basidiomata that is medium- to large-sized; cap and stipe are usually smooth, sometimes with appressed squama; hymenophore is veined with blunt, mostly strongly forking-anastomosing ridges, rarely smooth or nearly so; hyphal endings are mostly thick-walled; clamp connections are abundant everywhere (Buyck et al., 2014). In China, although several taxa of subg. *Cantharellus* have been described or reported in previous studies (Chiu, 1973; Shao et al., 2011, 2016b,^2021^; An et al., 2017; Cao et al., 2021; Zhang Y. Z. et al., 2021), many more undescribed novel species probably exist in the country. Recently, many collections of subg. *Cantharellus* in China have been collected, and they were studied using morphological and molecular phylogenetic analyses, aiming to (i) describe new taxa and (ii) elucidate the species diversity of subg. *Cantharellus* in China.

## Materials and Methods

### Morphological Studies

Field notes and digital photographs were made from fresh specimens. Specimens examined in this study were deposited in the Fungal Herbarium of Hainan Medical University (FHMU), Haikou City, Hainan Province of China. Macroscopic descriptions are based on the detailed notes and photographs taken from fresh basidiomata. Color codes follow Kornerup and Wanscher (1981). Samples were hand-sectioned and mounted in 5% KOH solution and 1% congo red. Sections of the pileipellis were cut radial-perpendicularly and halfway between the center and the margin of the pileus. The following notations [n/m/p] indicate that n basidiospores measured m basidiomata of p collections. Dimensions of basidiospores were presented in the form (a–)b–e–c(–d), where the range b–c contains at least 90% of the measured values, “a” and “d” were the extreme values, and “e” refers to the average length/width of basidiospores. Q refers to the length/width ratio of basidiospores; Q_*m*_ refers to the average Q of basidiospores and is given with standard deviation. For basidiospore shape, Q_*m*_ = 1.15–1.3 describes “broadly ellipsoid,” Q_*m*_ = 1.3–1.6 “ellipsoid,” and Q_*m*_ = 1.6–2.0 “elongate” (Yang, 2005). The terms referring to the size of basidioma are based on Bas (1969).

### Molecular Procedures

Total genomic DNA was obtained with the Plant Genomic DNA Kit (CWBIO, Beijing, China) according to the manufacturer’s instructions from collections dried with silica gel. Primer pairs used for amplification were as follows: nuc 28S rDNA D1-D2 domains (28S) with LR0R/LR5 (Vilgalys and Hester, 1990; James et al., 2006) and the translation elongation factor 1-α gene (*TEF1*) with EF1-α-F/EF1-α-R (Mikheyev et al., 2006). Polymerase chain reaction (PCR) conditions followed the program of Zhang Y. Z. et al. (2021). PCR products were checked in 1% (w/v) agarose gels. Amplified PCR products were sequenced using an ABI 3730 DNA Analyzer (Guangzhou Branch of BGI, China) with the same primers. Forward or reverse sequences were compiled with BioEdit (Hall, 1999). All sequences newly obtained in this study were deposited to GenBank^1^.

### Dataset Assembly

A total of sixty-nine DNA sequences (34 of 28S, and 35 of *TEF1*) from 36 collections were newly generated. Edited sequences were deposited in GenBank; the GenBank accession numbers are listed in Table 1. For the concatenated dataset, the sequences of 28S and *TEF1* from new collections were aligned with selected sequences of subg. *Cantharellus* from previous studies and GenBank (Table 1); *Cantharellus ibityensis* Buyck, Randrianj. and V. Hofst. was chosen as an outgroup inferred from Buyck et al. (2014). To test for phylogenetic conflict among 28S and *TEF1*, single-gene phylogenetic trees based on each of these two fragments were analyzed. The results of analyses showed that 28S and *TEF1* were not in conflict. Thus, two datasets (28S and *TEF1*) were aligned with MUSCLE v3.6 (Edgar, 2004) and manually optimized on BioEdit v7.0.9 (Hall, 1999); then, the two datasets were concatenated using Phyutility v2.2 for further analyses (Smith and Dunn, 2008).

**TABLE 1:** Taxa, vouchers, locations, and GenBank accession numbers of DNA sequences used in this study.

Taxon	Voucher	Locality	GenBank accession nos.		References
			28S	*TEF1*	
***Cantharellus albopileatus***	**N.K. **Zeng3026**** **(FHMU1987)**	**Hainan, Southern China**	**OM691480**	**OM811321**	**Present study**
***Cantharellus albopileatus***	**W.F. Lin7** **(FHMU6850)**	**Zhejiang, Eastern China**	**OM691481**	**OM811322**	**Present study**
*Cantharellus alborufescens*	BIO-Fungi 11689	Spain	KX828802	KX828832	Olariaga et al. (2017)
*Cantharellus altipes*	BB 07.019	United States	KF294627	GQ914939	Buyck et al. (2011, 2014)
*Cantharellus altipes*	BB 07.162	United States	KF294636	GQ914945	Buyck et al. (2011, 2014)
*Cantharellus altipes*	BB 07.005	United States	—	GQ914938	Buyck et al. (2011)
*Cantharellus altipes*	BB 07.055	United States	—	GQ914940	Buyck et al. (2011)
*Cantharellus altipes*	BB 07.112	United States	—	GQ914941	Buyck et al. (2011)
*Cantharellus amethysteus*	BB 07.284	Slovakia	KF294639	GQ914953	Buyck et al. (2011, 2014)
*Cantharellus amethysteus*	BB 07.309	Slovakia	KF294642	GQ914954	Buyck et al. (2011, 2014)
*Cantharellus anzutake*	C-2	Japan	LC085416	LC085470	Ogawa et al. (2017)
*Cantharellus anzutake*	C-84	Japan	LC085415	LC179800	Ogawa et al. (2017)
*Cantharellus applanatus*	PUN 3964	India	HM750918	—	Kumari et al. (2013)
*Cantharellus californicus*	OSC122878	United States	KX828795	KX828820	Olariaga et al. (2017)
*Cantharellus camphoratus*	12.09.06.av01	United States	KX592734	KX592735	Thorn et al. (2017)
*Cantharellus cascadensis*	OSC75917	United States	AY041158	—	Dunham et al. (2003)
*Cantharellus chicagoensis*	1073/JJMO-CANT-1	United States	—	KX857025	Buyck et al. (2016a)
***Cantharellus chuiweifanii***	**N.K. Zeng3474** **(FHMU5335)**	**Hainan, Southern China**	**OM717946**	**OM811340**	**Present study**
***Cantharellus chuiweifanii***	**N.K. Zeng4596** **(FHMU6846)**	**Hainan, Southern China**	**OM717947**	**OM811341**	**Present study**
***Cantharellus chuiweifanii***	**N.K. Zeng4559** **(FHMU6847)**	**Hainan, Southern China**	**OM717948**	**OM811342**	**Present study**
***Cantharellus chuiweifanii***	**N.K. Zeng2608** **(FHMU1703)**	**Hainan, Southern China**	**OM717949**	**OM811343**	**Present study**
***Cantharellus chuiweifanii***	**N.K. Zeng3452** **(FHMU5260)**	**Hainan, Southern China**	**OM717950**	**OM811344**	**Present study**
***Cantharellus chuiweifanii***	**N.K. Zeng4558** **(FHMU6848)**	**Hainan, Southern China**	**OM717951**	**OM811345**	**Present study**
***Cantharellus chuiweifanii***	**N.K. Zeng4897** **(FHMU6849)**	**Hainan, Southern China**	**OM717952**	**OM811346**	**Present study**
***Cantharellus chuiweifanii***	**N.K. Zeng1524** **(FHMU2412)**	**Hainan, Southern China**	**OM717953**	**OM811347**	**Present study**
***Cantharellus chuiweifanii***	**N.K. Zeng3463** **(FHMU2839)**	**Hainan, Southern China**	**OM717954**	**OM811348**	**Present study**
***Cantharellus chuiweifanii***	**N.K. Zeng5322** **(FHMU3236)**	**Hainan, Southern China**	**OM717955**	**OM811349**	**Present study**
***Cantharellus chuiweifanii***	**N.K. Zeng3392** **(FHMU5268)**	**Hainan, Southern China**	**OM717956**	**OM811350**	**Present study**
*Cantharellus cibarius*	WXH 2580	Jilin, NE China	—	KM893847	Unpublished
*Cantharellus cibarius*	BB 07.300	Slovakia	KF294641	GQ914950	Buyck et al. (2011, 2014)
*Cantharellus cibarius*	GE 07.025	France	KF294658	GQ914949	Buyck et al. (2011, 2014)
*Cantharellus cibarius*	WXH 2296	Finland	—	KM893842	Unpublished
*Cantharellus cibarius*	AFTOL-ID 607	Germany	—	DQ059050	Matheny et al. (2007)
*Cantharellus deceptivus*	1079/JJ NC-CANT-5	United States	—	KX857030	Buyck et al. (2016a)
*Cantharellus elongatipes*	PUN 3966	India	HM750929	—	Kumari et al. (2013)
*Cantharellus ferruginascens*	BB 07.283	Slovakia	KF294638	GQ914952	Buyck et al. (2011, 2014)
*Cantharellus ferruginascens*	BB 07.221	Slovakia	KF294637	GQ914951	Buyck et al. (2011, 2014)
*Cantharellus flavolateritius*	1078/JJ NC-CANT-4	United States	—	KX857029	Buyck et al. (2016a)
*Cantharellus flavolateritius*	1076/JJ NC-CANT-2	United States	—	KX857027	Buyck et al. (2016a)
*Cantharellus flavus*	C067	United States	—	JX030416	Foltz et al. (2013)
*Cantharellus formosus*	OSC 75930	United States	AY041164	—	Dunham et al. (2003)
*Cantharellus formosus*	OSC 76054	United States	AY041165	—	Dunham et al. (2003)
*Cantharellus hainanensis*	N.K. Zeng2289 (FHMU1931)	Hainan, Southern China	KY407524	KY407536	An et al. (2017)
*Cantharellus ibityensis*	BB 08.196	Madagascar	KF294650	GQ914980	Buyck et al. (2011, 2014)
*Cantharellus ibityensis*	BB 08.203	Madagascar	KF294651	JX192985	Buyck et al. (2013, 2014)
*Cantharellus indicus*	PUN 3962	India	HM750924	—	Kumari et al. (2013)
*Cantharellus iuventateviridis*	1542/SH13.7.2012	United States	—	KX857063	Herrera et al. (2018)
*Cantharellus iuventateviridis*	1543/SH14.7.2012	United States	—	KX857064	Herrera et al. (2018)
*Cantharellus laevihymeninus*	Yuan 13900	Yunnan, SW China	MW979520	MW999418	Cao et al. (2021)
*Cantharellus laevihymeninus*	Yuan 13902	Yunnan, SW China	MW979521	MW999419	Cao et al. (2021)
*“Cantharellus lateritius”*	PUN 3958	India	HM750919	—	Kumari et al. (2013)
*Cantharellus lateritius*	BB 06.319	United States	—	GQ914958	Buyck et al. (2011)
*Cantharellus lateritius*	BB 07.004	United States	—	GQ914955	Buyck et al. (2011)
*Cantharellus lateritius*	BB 07.025	United States	KF294628	GQ914957	Buyck et al. (2011, 2014)
*Cantharellus lateritius*	BB 07.058	United States	KF294633	GQ914959	Buyck et al. (2011, 2014)
*Cantharellus lewisii*	BB 02.197	United States	KF294623	GQ914961	Buyck et al. (2011, 2014)
*Cantharellus lewisii*	BB 07.003	United States	JN940597	GQ914962	Unpublished; Buyck et al. (2011)
*Cantharellus macrocarpus*	N.K. Zeng4036 (FHMU3303)	Hainan, Southern China	MT986060	MT990633	Zhang Y. Z. et al., 2021
*Cantharellus macrocarpus*	N.K. Zeng4050 (FHMU3304)	Hainan, Southern China	MT986061	MT990634	Zhang Y. Z. et al., 2021
***Cantharellus macrocarpus***	**N.K. *Zeng5235*** **(FHMU6385)**	**Hainan, Southern China**	**OM717945**	**OM811339**	**Present study**
*Cantharellus natarajanii*	PUN 3963	India	HM750926	—	Kumari et al. (2013)
*Cantharellus pallens*	AH44799	Spain	KR677537	KX828833	Olariaga et al. (2015, 2017)
*Cantharellus pallens*	AH39124	Morocco	KX828804	KX828834	Olariaga et al. (2017)
*Cantharellus pallens*	BB 09.441	Italy	KX907218	KX834411	De Kesel et al. (2016)
*Cantharellus pallens*	BB 09.430	Italy	KX907217	KX834410	De Kesel et al. (2016)
*Cantharellus persicinus*	1085/JJ MO-CANT-4	United States	—	KX857033	Buyck et al. (2016a)
*Cantharellus persicinus*	1685/MH 15.001	United States	—	KX857080	Buyck et al. (2016a)
*Cantharellus phasmatis*	C057	United States	JX030431	JX030417	Foltz et al. (2013)
*Cantharellus phasmatis*	C074	United States	—	JX030418	Foltz et al. (2013)
***Cantharellus pinetorus***	**N.K. Zeng4180** **(FHMU3759)**	**Hunan, Central China**	**OM691482**	**OM811323**	**Present study**
***Cantharellus pinetorus***	**N.K. Zeng4181** **(FHMU3749)**	**Hunan, Central China**	**OM691483**	**OM811324**	**Present study**
*Cantharellus pseudoformosus*	SMR-2009a	India	GU237071	—	Kumari et al. (2011)
*Cantharellus quercophilus*	BB 07.097	United States	KF294644	JX192981	Buyck et al. (2013, 2014)
***Cantharellus ravus***	**N.K. Zeng4176** **(FHMU6845)**	**Hunan, Central China**	**OM717944**	**OM811338**	**Present study**
*Cantharellus roseocanus*	CC29	United States	—	JX030415	Foltz et al. (2013)
*Cantharellus roseocanus*	DAOM220723	Canada	KX828810	KX828837	Olariaga et al. (2017)
*Cantharellus roseocanus*	DAOM220724	Canada	KX828811	KX828838	Olariaga et al. (2017)
*Cantharellus roseocanus*	MO 245717	—	—	MF784581	Unpublished
*Cantharellus roseofagetorum*	AH44786	Georgia	KX828813	KX828840	Olariaga et al. (2017)
*Cantharellus roseofagetorum*	AH44789	Georgia	KX828812	KX828839	Olariaga et al. (2017)
*Cantharellus* sp.	C-53	Japan	LC085421	LC085475	Ogawa et al. (2017)
*Cantharellus* sp.	C-141	Japan	LC085422	LC085476	Ogawa et al. (2017)
***Cantharellus* sp.**	**N.K. Zeng4113** **(FHMU3834)**	**Guangdong, Southern China**	**OM691493**	**OM811334**	**Present study**
***Cantharellus* sp.**	**N.K. Zeng3406** **(FHMU5266)**	**Hainan, Southern China**	**OM691494**	**OM811335**	**Present study**
***Cantharellus* sp.**	**S. Jiang106** **(FHMU4592)**	**Hainan, Southern China**	**OM691495**	**OM811336**	**Present study**
*Cantharellus* sp.	C-106	Japan	LC085418	LC085473	Ogawa et al. (2017)
*Cantharellus spectaculus*	C081	United States	JX030421	JX030414	Foltz et al. (2013)
*Cantharellus subalbidus*	OSC81782	United States	KX828814	KX828841	Olariaga et al. (2017)
*Cantharellus subamethysteus*	DS 06.218	Malaysia	KF294664	—	Buyck et al. (2014)
*Cantharellus subvaginatus*	1692	Korea	MG450678	—	Buyck et al. (2018)
*Cantharellus tenuithrix*	BB 07.035	United States	KF294629	GQ914946	Buyck et al. (2011, 2014)
*Cantharellus tenuithrix*	BB 07.125	United States	JN940600	GQ914947	Unpublished; Buyck et al. (2011)
*Cantharellus tenuithrix*	BB 14.099	United States	—	KX857054	Buyck et al. (2016a)
*“Cantharellus umbonatus”*	PUN 3968	India	HM750916	—	Kumari et al. (2013)
*Cantharellus vaginatus*	HKAS 55728	Yunnan, SW China	HM594680	—	Shao et al. (2011)
*Cantharellus vaginatus*	HKAS 55730	Yunnan, SW China	HM594681	—	Shao et al. (2011)
*Cantharellus vaginatus*	HKAS 55731	Yunnan, SW China	HM594682	—	Shao et al. (2011)
***Cantharellus vaginatus***	**LWF-1-1** **(FHMU6851)**	**Zhejiang, Eastern China**	**OM691484**	**OM811325**	**Present study**
***Cantharellus vaginatus***	**LWF-1-2** **(FHMU6852)**	**Zhejiang, Eastern China**	**OM691485**	**OM811326**	**Present study**
***Cantharellus vaginatus***	**M.S. Su201** **(FHMU6853)**	**Jiangxi, Eastern China**	**OM691486**	**OM811327**	**Present study**
***Cantharellus vaginatus***	**M.S. Su200** **(FHMU6855)**	**Jiangxi, Eastern China**	**OM691492**	**OM811333**	**Present study**
***Cantharellus vaginatus***	**N.K. Zeng3000** **(FHMU1961)**	**Hainan, Southern China**	**OM691487**	**OM811328**	**Present study**
***Cantharellus vaginatus***	**N.K. Zeng3009** **(FHMU1970)**	**Hainan, Southern China**	**OM691488**	**OM811329**	**Present study**
***Cantharellus vaginatus***	**N.K. Zeng2281** **(FHMU1533)**	**Hainan, Southern China**	**OM691489**	**OM811330**	**Present study**
***Cantharellus vaginatus***	**N.K. Zeng2521** **(FHMU1638)**	**Hainan, Southern China**	**OM691490**	**OM811331**	**Present study**
***Cantharellus vaginatus***	**Z.H. Chen MHHNU31942** **(FHMU6854)**	**Hunan, Central China**	**OM691491**	**OM811332**	**Present study**
*Cantharellus velutinus*	BB 14.038 (PC0142227)	United States	KX896789	KX857049	Buyck et al. (2016b)
*Cantharellus velutinus*	WR WV07.074	United States	—	KX857068	Buyck et al. (2016b)
*Cantharellus velutinus*	DM WV13.36	United States	—	KX857070	Buyck et al. (2016b)
*Cantharellus versicolor*	KUN-HKAS 55761	Yunnan, SW China	—	KM893856	Shao et al. (2016b)
*Cantharellus versicolor*	KUN-HKAS 58242	Yunnan, SW China	—	KM893857	Shao et al. (2016b)
*Cantharellus violaceovinosus*	Bandala 4513	Mexico	MF616524	MF616520	Herrera et al. (2018)
*Cantharellus violaceovinosus*	Corona 648	Mexico	MF616525	MF616521	Herrera et al. (2018)
*Cantharellus yunnanensis*	XieXD174	Yunnan, SW China	KU720333	KU720337	Unpublished
*Cantharellus yunnanensis*	ZhangJP117	Yunnan, SW China	KU720336	—	Unpublished
*Cantharellus yunnanensis*	SSC 1	Yunnan, SW China	—	KM893834	Shao et al. (2021)
*Cantharellus yunnanensis*	N.K. Zeng2778 (FHMU1767)	Hainan, Southern China	OK570080	OK562592	Tian et al. (2022)
***Cantharellus yunnanensis***	**N.K. Zeng2777** **(FHMU1766)**	**Yunnan, SW China**	**OM319633**	—	**Present study**
***Cantharellus yunnanensis***	**N.K. Zeng4084** **(FHMU3735)**	**Guangdong, Southern China**	**OM319632**	**OM811337**	**Present study**
***Cantharellus yunnanensis***	**N.K. Zeng5040** **(FHMU6841)**	**Yunnan, SW China**	**OM319634**	**OM321043**	**Present study**
***Cantharellus yunnanensis***	**N.K. Zeng3875** **(FHMU6842)**	**China**	**OM319635**	**OM321044**	**Present study**
***Cantharellus yunnanensis***	**Z.H. Chen MHHNU31318** **(FHMU6843)**	**Yunnan, SW China**	**OM319636**	**OM321045**	**Present study**
***Cantharellus yunnanensis***	**Z.H. Chen MHHNU32137** **(FHMU6844)**	**Hubei, Central China**	**OM319637**	**OM321046**	**Present study**
*Cantharellus yunnanensis*	Yuan 13983	China	MW979527	MW999428	Cao et al. (2021)
*Cantharellus yunnanensis*	Yuan 13985	China	MW979528	MW999429	Cao et al. (2021)
*Cantharellus yunnanensis*	Yuan 14539	China	MW979514	MW999422	Cao et al. (2021)
*Cantharellus yunnanensis*	Yuan 14636	China	MW979515	MW999423	Cao et al. (2021)

*GenBank numbers in bold indicate the newly generated sequences; SW Southwestern China, NE Northeastern China.*

### Phylogenetic Analyses

The combined nuclear dataset (28S + *TEF1*) was analyzed using maximum likelihood (ML) and Bayesian inference (BI). Maximum likelihood tree generation and bootstrap analyses were performed with the program RAxML7.2.6 (Stamatakis, 2006) running 1,000 replicates combined with an ML search. Bayesian analysis with MrBayes 3.1 (Huelsenbeck and Ronquist, 2005) implementing the Markov chain Monto Carlo (MCMC) technique and parameters predetermined with MrModeltes 2.3 (Nylander, 2004) was performed. The best-fit likelihood model of 28S and *TEF1* was GTR + I + G and SYM + I + G, respectively. Bayesian analysis of the combined nuclear dataset (28S + *TEF1*) was repeated for 20 million generations and sampled every 100 generations. Trees sampled from the first 25% of the generations were discarded as burn-in, and Bayesian posterior probabilities (PP) were then calculated for a majority consensus tree of the retained Bayesian trees. Runs were terminated once the average standard deviation of split frequencies went below 0.01.

## Results

### Molecular Data

**FIGURE 2:**
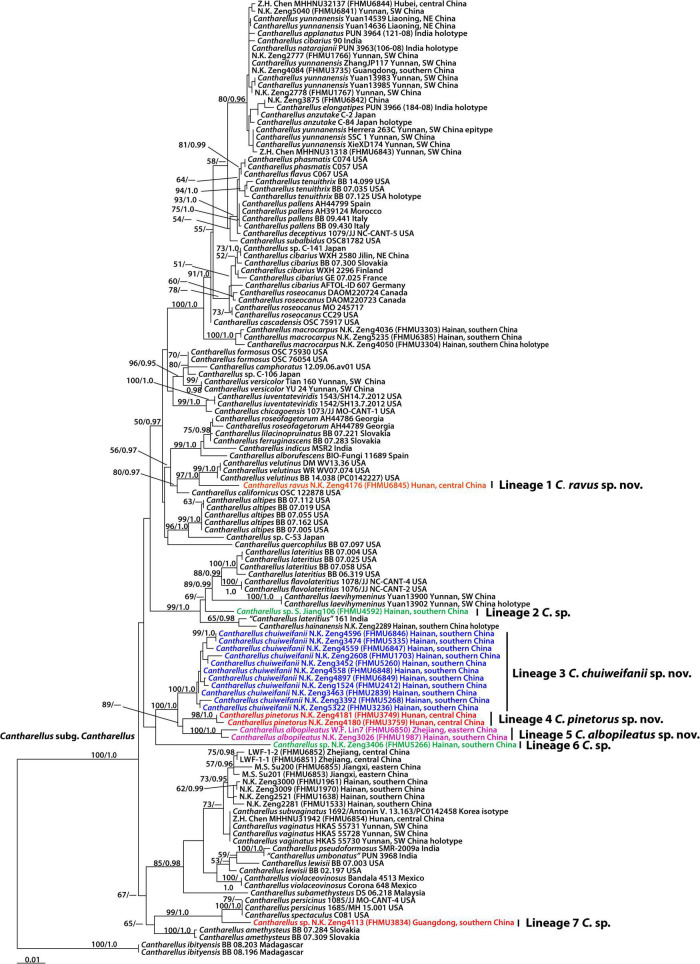
Phylogram inferred from a combined dataset (28S and *TEF1*) of the *Cantharellus* subg. *Cantharellus* using RAxML. RAxML likelihood bootstrap (BS ≥ 50%) and Bayesian posterior probabilities (PP ≥ 0.95) are indicated above or below the branches as RAxML BS/PP.

The combined dataset (28S + *TEF1*) of subg. *Cantharellus* consisted of 129 taxa and 1,707 nucleotide sites, and the alignment was submitted to TreeBase (S29413). The phylogram with branch lengths generated from RAxML and support values is shown in Figure 2. The topologies of the phylogenetic trees based on the combined dataset generated from ML and BI analyses were almost identical, but there was a slight variation in statistical support.

The present molecular data indicate that the Chinese species of subg. *Cantharellus* were grouped into fourteen independent lineages (Figure 2). A total of seven new lineages were identified in this study (Lineages 1–7 of Figure 2). Lineage 1 was comprised of one collection (FHMU6845) from central China; lineage 2 was comprised of one material (FHMU4592) from southern China; lineage 3, with strong statistical support (BS = 100%, PP = 1.0), was comprised of eleven collections (FHMU5335, FHMU6846, FHMU6847, FHMU1703, FHMU5260, FHMU6848, FHMU6849, FHMU2412, FHMU2839, FHM3236, and FHMU5268) from southern China; lineage 4, with high statistical support (BS = 98%, PP = 1.0), was comprised of two specimens (FHMU3749 and FHMU3759) both from central China; lineage 5, with strong statistical support (BS = 100%, PP = 1.0), was comprised of two materials (FHMU1987 and FHMU6850) from southern China and eastern China, respectively; lineage 6 was comprised of one collection (FHMU5266) from southern China; and lineage 7 was comprised of one specimen (FHMU3834) also from southern China (Figure 2).

### Taxonomy

***Cantharellus albopileatus*** N.K. Zeng, Y.Z. Zhang, and W.F. Lin, sp. nov.

Figures 3A,B, 4.

**FIGURE 3:**
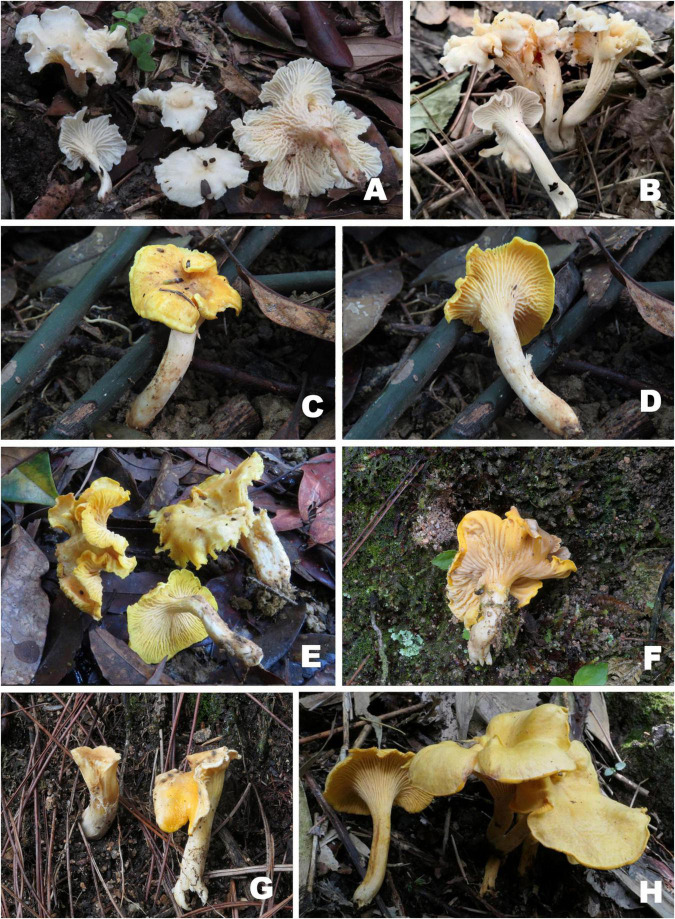
Basidiomata of *Cantharellus* subg. *Cantharellus* species. **(A,B)** *Cantharellus albopileatus* **(A)** FHMU1987, holotype; **(B)** FHMU6850; **(C–E)** *Cantharellus chuiweifanii* **(C,D)** FHMU5335; **(E)** FHMU2839; **(F,G)** *Cantharellus pinetorus* **(F)** FHMU3759, holotype; **(G)** FHMU3749; **(H)** *Cantharellus ravus* (FHMU6845, holotype). Photographs: **(A,C–H)** N. K. Zeng; **(B)** W. F. Lin.

**FIGURE 4:**
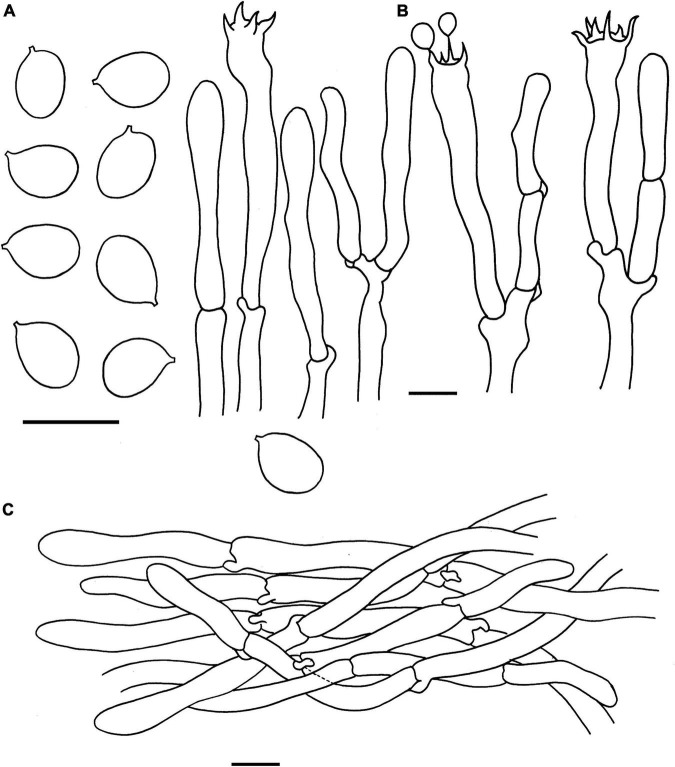
Microscopic features of *Cantharellus albopileatus* (FHMU1987, holotype). **(A)** Basidiospores. **(B)** Basidia. **(C)** Pileipellis. Scale bars = 10 μm. Drawings by Y. Z. Zhang.

MycoBank: MB843063

Diagnosis: It differs from other species of subg. *Cantharellus* by a cream to off-white basidioma, a well-developed hymenophore, and ellipsoid basidiospores.

Etymology: Latin “*albo-*” means white, “*pileatus*” means pileus, referring to the off-white pileus of our new species.

Holotype: China. Hainan Province: Yinggeling of Hainan Tropical Rainforest National Park, elev. 750 m, 28 May 2017, N. K. Zeng3026 (FHMU1987). GenBank accession number: 28S = OM691480, ITS = OM835804, *TEF1* = OM811321.

**Basidiomata** are very small to small-sized. **Pileus** is 2.5–5 cm in diameter and convex with a depressed center; the margin was strongly incurved, irregular, often wavy, and lobed; the surface is smooth, slightly greasy, and cream (3A1) to off-white (4A1) in color; the context above stipe was 0.3 cm in thickness, whitish (4A1), unchanging in color when injured. **Hymenophore** is composed of relatively well-developed, decurrent gill folds, branched to furcate, which becomes strongly intervened with age; these folds are about 0.1 cm broad and are yellowish (2A2) to white (4A1) in color. **Stipe** is 2–3 × 0.4–0.6 cm, central, subcylindrical, young solid, hollowing with age, and curved at the base; the surface was dry, whitish (4A1) to very pale cream (3A1), and nearly concolorous with hymenophore; the context is fleshy, firm, and whitish (3A1). **Taste** and **odor** is not distinctive. **Spore print** is not obtained.

**Basidiospores** [40/2/2] 6–7.18–8 × 5–5.46–6(–6.5) μm, *Q* = (1.09–)1.15–1.50(–1.6), Q_*m*_ = 1.32 ± 0.12, are ellipsoid, smooth, slightly thick-walled (0.5 μm), yellowish in KOH. **Basidia** is 43–65 × 5–10 μm, narrowly clavate, slightly thick-walled (up to 0.5 μm), 4-5-6-spored, and yellowish in KOH; sterigmata is 4–6 μm in length. **Cystidia** is absent. **Pileipellis** has a cutis that is 30–70 μm thick, is composed of mostly interwoven, cylindrical hyphae, is 6–12 μm wide, is thick-walled (up to 1 μm), and is faintly pale yellow in KOH; terminal cells are 21–108 × 5–10 μm in length, thin to slightly thick-walled (up to 0.5 μm), subcylindrical to subclavate, with obtuse apex. **Clamp connections** are present in all parts of basidioma.

Habitat: Solitary, scattered, or gregarious on the ground in forests dominated by fagaceous trees such as *Castanopsis fissa* (Champion ex Bentham) Rehder et E. H. Wilson.

Known distribution: southern and eastern China.

Other specimens were examined: China. Zhejiang Province: Hangzhou City, Tianmushan Nature Reserve, elev. 1100 m, 22 July 2020, W. F. Lin7 (FHMU6850).

Notes: *Cantharellus albus* S. P. Jian and B. Feng, originally described from Yunnan, southwestern China, has a white basidioma. However, by presenting the features such as a stipe turning yellow when bruised and a lower value of Q_*m*_, it is a member of subg. *Parvocantharellus* Eyssart. and Buyck (Jian et al., 2020). The white basidioma makes *Cantharellus albopileatus* somewhat similar to the American *Cantharellus subalbidus* A. H. Sm. and Morse; however, this latter species in addition to being associated with trees of Pinaceae (Smith and Morse, 1947) possesses larger basidioma [pileus 5–10 (–14) cm], and it is also distinctly differentiated at the molecular level (Figure 2).

In the phylogenetic analyses, *Cantharellus albopileatus* forms a well-supported (BS = 100%, PP = 1.0) monophyletic clade, together with the newly described *Cantharellus chuiweifanii*, equally a tropical species, and *Cantharellus pinetorus* from central China (Figure 2), which is quite different from any of the other northern hemisphere species in subg. *Cantharellus.* However, *Cantharellus pinetorus* is associated with trees of Pinaceae (refer to the descriptions under *Cantharellus pinetorus*); both *Cantharellus pinetorus* and *Cantharellus chuiweifanii* have a yellow pileus (refer to the descriptions under *Cantharellus chuiweifanii*).

***Cantharellus chuiweifanii*** N.K. Zeng, Y.Z. Zhang, and Zhi Q. Liang, sp. nov.

Figures 3C–E, 5.

**FIGURE 5:**
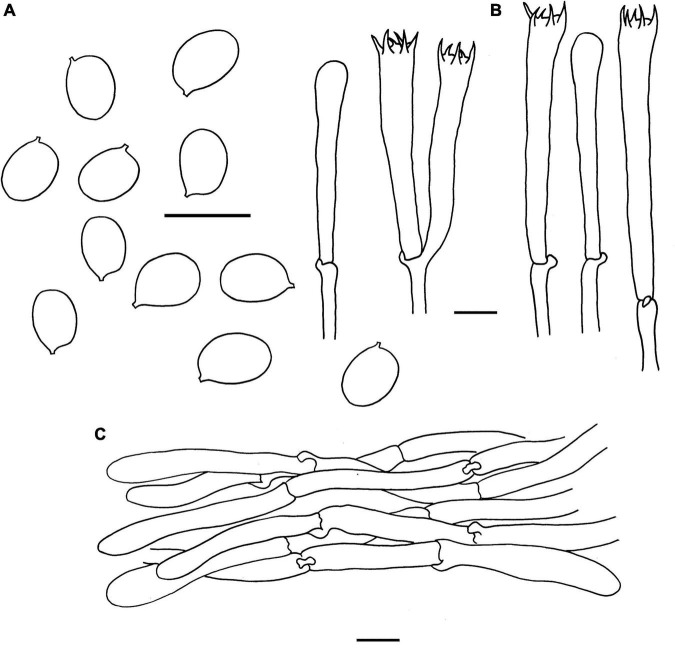
Microscopic features of *Cantharellus chuiweifanii* (FHMU2412, holotype). **(A)** Basidiospores. **(B)** Basidia. **(C)** Pileipellis. Scale bars = 10 μm. Drawings by Y. Z. Zhang and D. Y. An.

MycoBank: MB843062

Diagnosis: It differs from other species of subg. *Cantharellus* by an egg-yolk yellow to bright yellow, shiny pileus, a whitish stipe, a well-developed hymenophore, ellipsoid basidiospores, and a distribution in tropical Asia.

Etymology: Latin “*chuiweifanii*” is named after Chinese mycologist W. F. Chiu; for that, he published the first new species of *Cantharellus* in China, i.e., *Cantharellus yunnanensis*.

Holotype: China. Hainan Province: Limushan of Hainan Tropical Rainforest National Park, elev. 650 m, 11 May 2014, N. K. Zeng1524 (FHMU2412). GenBank accession number: 28S = OM717953, *TEF1* = OM811347.

**Basidiomata** are very small to medium-sized. **Pileus** is 2–5 cm in diameter and plano-convex at first but soon depressed in the center; the margin was first very regular and strongly incurved and then became more wavy, the surface is smooth, pale yellow, egg-yolk yellow (1A5) to bright yellow (1A7), sometimes tinged with brownish, and shiny in color; the context above stipe was 0.05–0.15 cm in thickness, yellowish (3A3) to orange yellow (3A8), but unchanging in color when injured. **Hymenophore** is composed of well-developed, decurrent, well-spaced, and unequal gill folds, especially near the extreme cap margin with many very short lamellulae or also often forked; these folds are 0.1–0.2 cm broad and are pale yellow, egg-yolk yellow (1A4) to orange yellow in color (2A7). **Stipe** is 1.2–4 × 0.4–1.0 cm, central, cylindrical; surface dry, whitish (5A1), or yellowish-brown (1A2) in color; the context is fleshy, firm, and yellowish (2A2). **Taste** and **odor** is not distinctive. **Spore print** is not obtained.

**Basidiospores** [173/18/11] 6–7.05–8 × 4.5–5.03–5.5(–6) μm, *Q* = (1.18–)1.27–1.60(–1.67), Q_*m*_ = 1.41 ± 0.10, are ellipsoid, smooth, slightly thick-walled (0.5 μm), yellowish in KOH. **Basidia** is –57-67 × 8-10 long, narrow, subcylindric, slightly thick-walled (0.5–0.7 μm), 4-5-6-spored, yellowish in KOH; sterigmata is 5–7 μm in length. **Cystidia** is absent. **Pileipellis** has a cutis that is 70–180 μm thick, is composed of mostly interwoven, cylindrical hyphae, is 5–8 μm wide, is slightly thick-walled (0.5–0.8 μm), and is faintly pale yellow in KOH; terminal cells are 52–148 × 4–7 μm in length, slightly thick-walled (0.5–0.8 μm), subcylindrical to subclavate, with obtuse apex. **Clamp connections** are present in all parts of basidioma.

Habitat: Solitary, scattered, or gregarious on the ground in forests dominated by fagaceous trees such as *Lithocarpus* spp.

Known distribution: southern China.

Other specimens were examined: China. Hainan Province: Yinggeling of Hainan Tropical Rainforest National Park, elev. 750 m, 5 August 2015, N. K. Zeng2608 (FHMU1703); Jianfengling of Hainan Tropical Rainforest National Park, elev. 850 m, 27 June 2018, N. K. Zeng3392 (FHMU5268); same location, 28 June 2018, N. K. Zeng3452, 3463, 3474 (FHMU5260, FHMU2839, FHMU5335); same location, 10 August 2020, N. K. Zeng4558, 4559 (FHMU6848, FHMU6847); same location, 11 August 2020, N. K. Zeng4596 (FHMU6846); same location, 29 July 2021, N. K. Zeng5322 (FHMU3236); Wanning County, Bofangling, elev. 70 m, 29 August 2020, N. K. Zeng4897 (FHMU6849).

Notes: *Cantharellus chuiweifanii* looks like *Cantharellus subcibarius* Corner and *Cantharellus yunnanensis.* However, *Cantharellus subcibarius* has a yellow stipe, a context turning yellow to orange-brown when bruised, and larger basidiospores [(7.3–)7.5–7.89–8.3(–8.5) × (5.2–)5.6–6.10–6.5(–6.9) μm, *Q* = (1.12–)1.23–1.30–1.36(–1.40)] (Corner, 1966; Buyck et al., 2021); *Cantharellus yunnanensis* has a yellow stipe, wider basidiospores measuring (6.5–)7–8(–8.5) × 5–6(–6.5) μm (Shao et al., 2021), and it is also distinctly differentiated at the molecular level (Figure 2). *Cantharellus chuiweifanii* is phylogenetically associated with *Cantharellus albopileatus* and *Cantharellus pinetorus* (Figure 2), and the differences in the three taxa have been discussed under *Cantharellus albopileatus*.

***Cantharellus pinetorus*** N.K. Zeng, Y.Z. Zhang and Zhi Q. Liang, sp. nov.

Figures 3F,G, 6.

**FIGURE 6:**
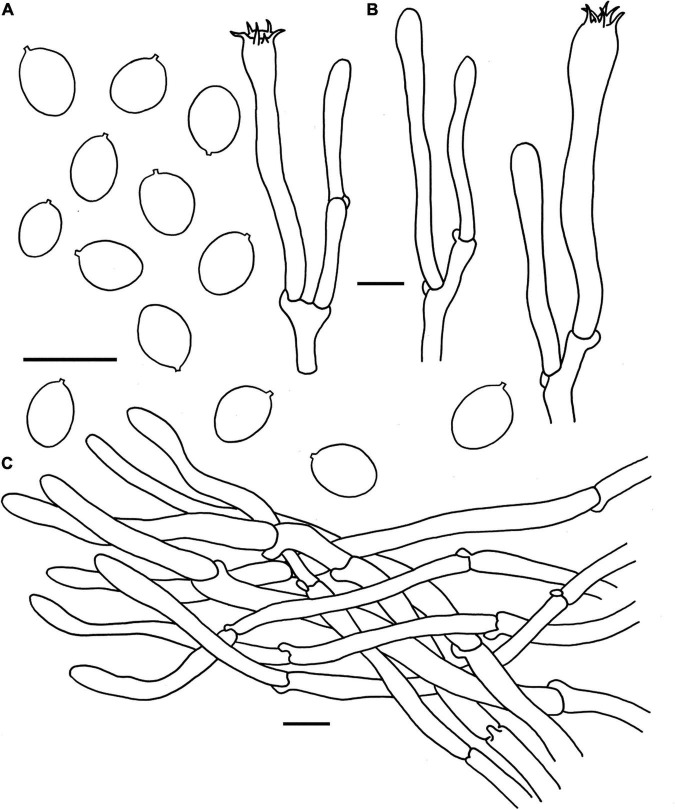
Microscopic features of *Cantharellus pinetorus* (FHMU3759, holotype). **(A)** Basidiospores. **(B)** Basidia. **(C)** Pileipellis. Scale bars = 10 μm. Drawings by Y. Z. Zhang.

MycoBank: MB843064

Diagnosis: It differs from other species of subg. *Cantharellus* by a bright yellow to orange-yellow pileus, a cream to grayish yellow stipe, a well-developed hymenophore, broadly ellipsoid to ellipsoid basidiospores, and it is associated with pine trees.

Etymology: Latin “*pinetorus*” refers to the association of the new species with pine forests.

Holotype: China. Hunan Province: Yizhang County, Mangshan National Nature Reserve, elev. 750 m, 30 July 2019, N. K. Zeng4180 (FHMU3759). GenBank accession number: 28S = OM691482, *TEF1* = OM811323.

**Basidiomata** are small to medium-sized. **Pileus** is 3.5–5 cm in diameter and convex when young and then applanate with depressed center; the surface is smooth, bright yellow (3A5) to orange-yellow in color (3A6); the margin is incurved and irregularly wavy; the context above stipe is 0.35–0.55 cm in thickness, yellow (3A7), but unchanging in color when injured. **Hymenophore** is veined and decurrent; these folds are 0.05–0.2 cm broad and are lemon yellow (1A6) to pale yellow in color (4A2). **Stipe** is 2.5–3 × 0.5–0.6 cm, cylindrical, central, and hollow; the surface is dry and cream (4A1) to grayish yellow (4A2) in color; the context is white (3A2). **Taste** and **odor** is not distinctive. **Spore print** is not obtained.

**Basidiospores** [40/2/2] 6–6.98–7.5(–8) × 5–5.36–6 μm, *Q* = (1.09–)1.17–1.5, Q_*m*_ = 1.30 ± 0.09, are broadly ellipsoid to ellipsoid, smooth, slightly thick-walled (0.5 μm), yellowish in KOH. **Basidia** is 57–68 × 4–10 μm, long, narrow, subcylindric, slightly thick-walled (up to 0.5 μm), 3-4-5-spored, and yellowish in KOH; sterigmata is 2–4 μm in length. **Cystidia is** absent. **Pileipellis** has a cutis that is 100–150 μm thick, is composed of mostly interwoven, cylindrical hyphae, is 6–9 μm wide, is slightly thick-walled (up to 0.5 μm), and is faintly pale yellow in KOH; terminal cells are 40–61 × 4–6 μm in length, slightly thick-walled (0.5–0.7 μm), subcylindrical to subclavate, with obtuse apex. **Clamp connections** are present in all parts of basidioma.

Habitat: Solitary, scattered, or gregarious on the ground, in forests dominated by *Pinus massoniana* Lamb.

Known distribution: central China.

Other specimens were examined: China. Hunan Province: Yizhang County, Mangshan National Nature Reserve, elev. 750 m, 30 July 2019, N. K. Zeng4181 (FHMU3749).

Notes: Malaysian *Cantharellus ianthinus* Corner and *Cantharellus subcibarius* are morphologically similar to *Cantharellus pinetorus*. However, *Cantharellus ianthinus* has purple fibrils on the surfaces of the pileus and stipe, and larger basidiospores measuring 8–10.5 × 5.5–7 μm (Corner, 1966); *Cantharellus subcibarius* has a context turning yellow to orange-brown when bruised, and it is not associated with trees of Pinaceae (Corner, 1966; Buyck et al., 2021). In the phylogenetic analyses, *Cantharellus pinetorus* is allied with *Cantharellus albopileatus* and *Cantharellus chuiweifanii* (Figure 2), the differences in the three taxa have been discussed under *Cantharellus albopileatus*.

***Cantharellus ravus*** N.K. Zeng, Y.Z. Zhang, and Zhi Q. Liang, sp. nov.

Figures 3H, 7.

**FIGURE 7:**
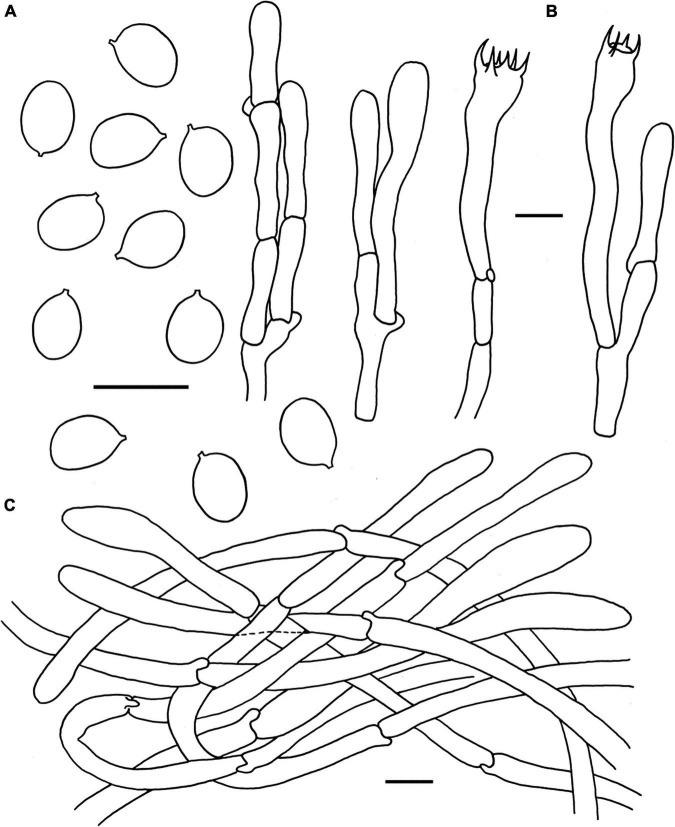
Microscopic features of *Cantharellus ravus* (FHMU6845, holotype). **(A)** Basidiospores. **(B)** Basidia. **(C)** Pileipellis. Scale bars = 10 μm. Drawings by Y. Z. Zhang.

MycoBank: MB843065

Diagnosis: It differs from other species of subg. *Cantharellus* by a yellowish to grayish yellow, dull pileus, a well-developed hymenophore, and ellipsoid basidiospores.

Etymology: Latin “*ravus*” refers to the grayish yellow basidioma of our new species.

Holotype: China. Hunan Province: Yizhang County, Mangshan National Nature Reserve, elev. 550 m, 29 July 2019, N. K. Zeng4176 (FHMU6845). GenBank accession number: 28S = OM717944, *TEF1* = OM811338.

**Basidiomata** are small to medium-sized. **Pileus** is 3.5–8 cm in diameter and plano-convex to infundibuliform; the surface is smooth, yellowish (1A2) to grayish yellow (2A4) in color, and dull; the margin is incurved or downward; the context above stipe is about 0.3 cm in thickness, yellowish (3A3), unchanging in color when injured. **Hymenophore** is veined and decurrent; these folds are about 0.1 cm high, forking, creamy yellow (1A4) to yellowish (3A2) in color. **Stipe** is 3–4 × 0.6–0.9 cm, central, cylindrical; surface dry, grayish yellow to fulvous (4A4), but whitish (5A1) at base. **Taste** and **odor** is not distinctive. **Spore print** is not obtained.

**Basidiospores** [37/2/1] (6–)6.5–7.04–7.5 × 4.5–5.24–5.5 μm, *Q* = (1.18–)1.2–1.5(–1.56), Q_*m*_ = 1.35 ± 0.08, are ellipsoid, smooth, slightly thick-walled (0.5 μm), yellowish in KOH. **Basidia** is 45–63 × 8–9 μm, long, narrow, subcylindric, slightly thick-walled (up to 0.5 μm), 4-5-6 spored, yellowish in KOH; sterigmata is 6–8 μm in length. **Cystidia** is absent. **Pileipellis** has a cutis composed of mostly cylindrical, that is 5–12 μm wide, is slightly thick-walled (up to 1 μm) hyphae, and is faintly pale yellow in KOH; terminal cells are 45–82 × 6–10 μm, slightly thick-walled (0.5–0.7 μm), subcylindrical to subclavate, with obtuse apex. **Clamp connections** are present in all parts of basidioma.

Habitat: Gregarious on the ground in forests dominated by fagaceous trees such as *Lithocarpus* spp.

Known distribution: central China.

Notes: *Cantharellus subcibarius* and *Cantharellus yunnanensis* are also morphologically similar to our new species. However, both *Cantharellus subcibarius* and *Cantharellus yunnanensis* have shiny pileal surfaces. Moreover, *Cantharellus subcibarius* has a context turning yellow to orange-brown when bruised, and larger basidiospores [(7.3–)7.5–7.89–8.3(–8.5) × (5.2–)5.6–6.10–6.5(–6.9) μm, *Q* = (1.12–)1.23–1.30–1.36(–1.40)] (Corner, 1966; Buyck et al., 2021); *Cantharellus yunnanensis* also has larger basidiospores measuring (6.5–) 7–8 (–8.5) × 5–6 (–6.5) μm (Shao et al., 2021), and it is distinctly differentiated at the molecular level (Figure 2). Our molecular data also indicated that *Cantharellus ravus* is closely related to the North American *Cantharellus californicus* D. Arora and Dunham and *Cantharellus velutinus* Buyck and V. Hofst (Figure 2). However, *Cantharellus californicus* has much larger basidiospores measuring 7–9.30–12 μm × 5–6.45–8 μm (Arora and Dunham, 2008); *Cantharellus velutinus*, a very variable species with yellow to pink fruiting bodies and pubescent pileus surface, has longer but narrower basidiospores measuring (6.7–)7.3–7.84–8.4(–9.2) × (3.7–)4.2–4.61–5.0(–5.2) μm and hyphal extremities of the pileipellis with conspicuously thickened cell walls (Buyck et al., 2016b).

## Discussion

### Morphological Features and Hosts of *Cantharellus* Subgenus *Cantharellus*

In agreement with the previous hypotheses (Buyck et al., 2014), most species in China have nearly glabrous pileus, with the exception of *Cantharellus vaginatus* S. C. Shao, X. F. Tian, and P. G. Liu and *Cantharellus versicolor* S. C. Shao and P. G. Liu having a squamulose cap (Shao et al., 2011, 2016b). As noted by Buyck et al. (2014), most taxa in subg. *Cantharellus* have yellow pileus. In China, *Cantharellus chuiweifanii*, *Cantharellus cibarius* Fr, *Cantharellus hainanensis* N. K. Zeng, Zhi Q. Liang, and S. Jiang, *Cantharellus laevihymeninus* T. Cao and H. S. Yuan, *Cantharellus macrocarpus* N. K. Zeng, Y. Z. Zhang, and Zhi Q. Liang, *Cantharellus pinetorus*, *Cantharellus ravus*, *Cantharellus vaginatus*, and *Cantharellus yunnanensis* also have pilei colored with yellow; *Cantharellus albopileatus* is characterized with white pileus; squamules on pileal surface of *Cantharellus vaginatus* are fulvous to brown (Shao et al., 2011), and sandy brown to dark brown in *Cantharellus versicolor* was observed (Shao et al., 2016b).

Besides macro-morphology, some micro-morphological features can also be used to discriminate subg. *Cantharellus* species. For example, species of the subgenus usually have abundant clamps, and hyphal endings in pileipellis are mostly thick-walled (Buyck et al., 2014). Our four new species and previous taxa of subg. *Cantharellus* in China also possess clamp connections and thick-walled hyphal endings in pileipellis (Shao et al., 2011, 2016b,^2021^; An et al., 2017; Cao et al., 2021; Zhang Y. Z. et al., 2021).

In regard to ecological preference, many species such as *Cantharellus altipes* Buyck and V. Hofst, *Cantharellus macrocarpus*, and *Cantharellus tenuithrix* Buyck and V. Hofst were reported to grow in pine-oak woods (Buyck and Hofstetter, 2011; Shao et al., 2021; Zhang Y. Z. et al., 2021). In China, *Cantharellus cibarius*, *Cantharellus vaginatus*, and *Cantharellus yunnanensis* were also reported to grow in pine-oak forests (Shao et al., 2011, 2021); *Cantharellus albopileatus*, *Cantharellus chuiweifanii*, *Cantharellus hainanensis*, and *Cantharellus ravus* grow in forests dominated by fagaceous trees, whereas *Cantharellus pinetorus* and *Cantharellus versicolor* are associated with trees of Pinaceae (Shao et al., 2011, 2016b; An et al., 2017).

Most species of subg. *Cantharellus* sections *Cantharellus* and *Amethystini* Buyck and V. Hofstetter have well-developed hymenophore, section *Sublaeves* Buyck and V. Hofstetter harbors taxa with smooth hymenophore, and section *Amethystini* Buyck and V. Hofstetter usually has a pileus with appressed squama. Judging from the positions of our new species and Chinese previous taxa in the molecular phylogenetic tree (Figure 2), plus the morphological features, *Cantharellus cibarius*, *Cantharellus macrocarpus*, *Cantharellus ravus*, *Cantharellus versicolor*, and *Cantharellus yunnanensis* are the members of section *Cantharellus*, *Cantharellus hainanensis*, and *Cantharellus laevihymeninus*, also have ill-developed hymenophore (An et al., 2017; Cao et al., 2021), and belong to the section *Sublaeves*. *Cantharellus vaginatus* is a member of section *Amethystini*, and *Cantharellus albopileatus*, *Cantharellus chuiweifanii*, and *Cantharellus pinetorus* probably represent a new section, which will be further studied in the future.

### Species Diversity of *Cantharellus* Subgenus *Cantharellus*

High species diversity of subg. *Cantharellus* in China was revealed in this study, and fourteen lineages were identified (Figure 2). A total of four (lineages 1, 3–5 of Figure 2) were here described as new species: *Cantharellus albopileatus*, *Cantharellus chuiweifanii*, *Cantharellus pinetorus*, and *Cantharellus ravus*; others represent previously described taxa: *Cantharellus cibarius*, *Cantharellus hainanensis*, *Cantharellus laevihymeninus*, *Cantharellus macrocarpus*, *Cantharellus vaginatus*, *Cantharellus versicolor*, and *Cantharellus yunnanensis*, whereas three lineages (lineages 2, 6–7 of Figure 2) remain still undescribed.

Up to now, taxa in subg. *Cantharellus* are all from northern hemisphere (Buyck et al., 2014). Most species of the subgenus included in the present dataset are from temperature areas of northern hemisphere including North America and Europe (Figure 2). In China, with the exception of *Cantharellus cibarius* and *Cantharellus versicolor* in temperate areas, most species, viz. *Cantharellus albopileatus*, *Cantharellus chuiweifanii*, *Cantharellus hainanensis*, *Cantharellus macrocarpus*, *Cantharellus pinetorus*, *Cantharellus ravus*, *Cantharellus vaginatus*, and *Cantharellus yunnanensis*, occur in subtropical or tropical China. With more field investigations, more taxa of the subgenus will be uncovered in the subtropical and tropical regions.

### Phylogenetic Relationships and Geographic Divergence of Chinese *Cantharellus* Subgenus *Cantharellus*

Our molecular data based on two-locus DNA sequences (28S + *TEF1*) with many new specimens from China have contributed to our knowledge of subg. *Cantharellus*.

It is clear that there are several clades having taxa from both sides of the Pacific, and allied species from East Asia and North America are obviously inferred from this molecular phylogenetic tree (Figure 2). For example, Chinese *Cantharellus versicolor* is related to North American *Cantharellus camphoratus* R. H. Petersen and *Cantharellus formosus* Corner; our new species *Cantharellus ravus* is affiliated with *Cantharellus californicus* and *Cantharellus velutinus*, two species both described from United States; one collection tentatively named *C.* sp. (FHMU3834) appears closely related to North American-type collection of *Cantharellus spectaculus* Foltz and T. J. Volk (Figure 2), which is an earlier synonym of *Cantharellus persicinus* Petersen (Buyck et al., 2016c). Our study did not identify disjunct populations of the same purported taxon in the two areas (Figure 2). Similar scenarios have been documented for many other fungi (Zhang et al., 2004; Zeng et al., 2013, 2016).

Biogeographic connections between East Asia and Europe have also been discussed in other fungi such as *Amanita* Pers., *Phylloporus* Quél. and *Rhodotus* Maire (Zhang et al., 2004; Zeng et al., 2013; Tang et al., 2014). In this study, we found that *Cantharellus cibarius* occurs in northeastern China and Europe (Figure 2).

In addition, we also noted that *Cantharellus hainanensis* is associated with one specimen labeled as *Cantharellus lateritius* from India (Figure 2). Besides northeastern China and Europe, the geographical distribution range of *Cantharellus cibarius* also extends to Japan (Figure 2).

## Data Availability Statement

The data presented in the study are deposited in the https://www.ncbi.nlm.nih.gov/GenBank and https://www.mycobank.org/page/Home/MycoBank repository, accession number of GenBank (28S: OM319632-OM319637, OM691480-OM691495, OM717944-OM717956; TEF1: OM321043-OM321046, OM811321-OM811350; and ITS: OM835791-OM835808) and MycoBank (MB843062-MB843065).

## Author Contributions

Z-QL and N-KZ contributed to the conceptualization. Y-ZZ performed the methodology, wrote the original draft preparation, and carried out the formal analysis. Y-ZZ and D-YA performed the experiment. N-KZ, W-FL, M-SS, Z-HC, PZ, and SJ carried out the resources. N-KZ, BB, and Z-QL wrote, reviewed, and edited the manuscript. N-KZ and Z-QL supervised the data. N-KZ carried out the project administration and funding acquisition. All authors contributed to the article and approved the submitted version.

## Conflict of Interest

The authors declare that the research was conducted in the absence of any commercial or financial relationships that could be construed as a potential conflict of interest.

## Publisher’s Note

All claims expressed in this article are solely those of the authors and do not necessarily represent those of their affiliated organizations, or those of the publisher, the editors and the reviewers. Any product that may be evaluated in this article, or claim that may be made by its manufacturer, is not guaranteed or endorsed by the publisher.

## Footnotes

1
https://www.ncbi.nlm.nih.gov/genbank
